# Supplementary material for: Pulsed-Xenon Ultraviolet Light Highly Inactivates Human Coronaviruses on Solid Surfaces, Particularly SARS-CoV-2
Source: Int J Environ Res Public Health. 2022 Oct 23;19(21):13780. doi: 10.3390/ijerph192113780 (PMC9653743; doi:10.3390/ijerph192113780)
Supplement: Supplementary file 1 [file ijerph-19-13780-s001.zip › ijerph-1964840-supplementary.pdf]

Supplementary Table S1. Comparison of doses of UVC light emitted by PX-UV source at 1.5 m and conventional UV lamp at 2 m

Light Strike™ Germ-Zapping™ Robot - 1.5 m		TUV PI-L 36W, UV-C – 2 m	
**Time (s)**	**Dose (mJ/cm^2)**	**Time (s)**	**Dose (mJ/cm^2)**
30	0.717	30	0.704
60	1.391	60	1.409
90	2.060	90	2.113
120	2.728	120	2.818
150	3.398	150	3.521
180	4.067	180	4.226
210	4.737	210	4.930
240	5.402	240	5.634
270	6.068	270	6.338
300	6.734	300	7.043

Supplementary Table S2. Irradiance data measured with StellarNet Blue-Wave Spectroradiometer at 1.5 and 2 m.

**Light Strike™ Germ-Zapping™ Robot**	**1.5 m**	
	**μW/cm²**	**U (μW/cm²)**
**UVC**	22.23	0.87
**UVB**	15.52	0.60
**UVA**	49.50	1.20
**Total UV light**	87.10	1.60
**TUV PI-L 36W, UV-C**	**2.0 m**	
	**μW/cm²**	**U (μW/cm²)**
**UVC**	23.48	4.35

U, measurement unceraint y.

Supplementary Table S3. Dose data of Light Strike™ Germ-Zapping™ Robot calculated at 1.5 m from 30 to 300 seconds of exposure

	Dose (mJ/cm^2) - 1.5 m			
**Time (s)**	**UVC**	**UVB**	**UVA**	**TOTAL UV**
30	0.717	0.501	1.597	2.810
60	1.391	0.971	3.096	5.448
90	2.060	1.438	4.586	8.070
120	2.728	1.904	6.072	10.685
150	3.398	2.372	7.565	13.312
180	4.067	2.839	9.055	15.933
210	4.737	3.307	10.545	18.555
240	5.402	3.771	12.027	21.162
270	6.068	4.236	13.509	23.770
300	6.734	4.701	14.993	26.381

Supplementary Table S4. Dose data of TUV PI-L 36W, UV-C calculated at 2 m from 30 to 300 seconds of exposure

Time (s)	Dose (mJ/cm^2) – 2 m
30	0.704
60	1.409
90	2.113
120	2.818
150	3.521
180	4.226
210	4.930
240	5.634
270	6.338
300	7.043
